# Investigating the Knowledge, Attitudes, and Practice of Family Caregivers in Post‐Hip Fracture Surgery Care: A Descriptive‐Analytical Study

**DOI:** 10.1155/tswj/3033303

**Published:** 2026-01-28

**Authors:** Esmaeil Fakharian, Mojtaba Sehat, Azam Jahangirimehr, Mohammad Reza Fazel, Mehrdad Mahdian, Khadijeh Kalanfarmanfarma, Alireza AkbarzadehArab, Masoumeh Abedzadeh-Kalahroudi, Masoomeh Vaeidi, Soudabeh Yarmohammadi, Fahimeh Sarbandi

**Affiliations:** ^1^ Trauma Research Center, Department of Neurosurgery, Kashan University of Medical Sciences, Kashan, Iran, kaums.ac.ir; ^2^ Trauma Research Center, Department of Community Medicine, Faculty of Medicine, Kashan University of Medical Sciences, Kashan, Iran, kaums.ac.ir; ^3^ Shoushtar Faculty of Medical Sciences, Shoushtar, Iran; ^4^ Trauma Research Center, Kashan University of Medical Sciences, Kashan, Iran, kaums.ac.ir; ^5^ Department of Orthopedic Surgery, Kashan University of Medical Sciences, Kashan, Iran, kaums.ac.ir; ^6^ Trauma Nursing Research Center Kashan University of Medical Sciences, Kashan, Iran

**Keywords:** attitudes, caregivers, health knowledge, hip fractures, home nursing, postoperative care, practice

## Abstract

**Background:**

Hip surgery is a critical medical procedure that necessitates specialized postoperative care. The knowledge, attitudes, and practice of family caregivers are pivotal in ensuring patient recovery and minimizing complications. This study is aimed at exploring these three key aspects among caregivers.

**Methods:**

This descriptive‐analytical study included 215 family caregivers selected through convenience sampling from Shahid Beheshti Hospital in Kashan. The data collection tool was a researcher‐developed questionnaire consisting of four sections: demographic characteristics, knowledge, attitudes, and practice (KAP). Data analysis was performed using SPSS Version 26, employing descriptive statistics (mean and standard deviation) and inferential statistics (Pearson correlation, independent *t*‐test, ANOVA, and linear regression).

**Results:**

Among the caregivers, 69.8% were female and 30.2% were male, with the majority (41.9%) aged between 42 and 51 years, and 33.5% holding a diploma. The mean attitude score (84.82 ± 5.22) was higher than both the practice score (78.65 ± 5.14) and the knowledge score (74.68 ± 6.29). The strongest and most direct correlation was found between practice and knowledge (*p* < 0.001, *R* = 0.422). The variables of knowledge, Iranian nationality, and caregiving history had the most significant impact on improving caregiving behavior, collectively accounting for 31% of the behavioral changes (*R* ^2^ = 0.312).

**Conclusion:**

Enhancing caregiver knowledge, along with factors such as Iranian nationality and caregiving history, plays a crucial role in improving patient care behavior. It is recommended to develop targeted training programs for inexperienced and non‐Iranian caregivers to enhance the quality of care provided.

## 1. Introduction

Hip fracture is a significant orthopedic injury that poses a serious threat to human health and is highly prevalent [1]. This type of fracture imposes a substantial burden on individuals and society, driven by high treatment costs, diminished health‐related quality of life, and reduced survival rates [2]. Projections suggest that by 2050, the global incidence of hip fractures will rise by 240% in women and 310% in men [3]. The risk of hip fracture varies considerably across different regions of the world. This variation is primarily influenced by factors such as age, sex, race, geographic region, and environment. Additional contributing factors include obesity, the general health status of the population, access to preventive and treatment services, as well as genetic and behavioral factors [4, 5].

The mortality rate associated with hip fractures in patients over 65 years of age is reported to be 15 times higher than that in younger patients [6]. Various studies have identified risk factors such as age [7, 8], gender [8, 9], fracture type [9], and walking ability [8]. Given that certain factors like age, gender, and fracture type are nonmodifiable, it is essential to focus on preventable factors such as residential status, smoking, and conditions like cardiovascular disease, pulmonary disease, and diabetes [10].

Patients with hip fractures frequently undergo multiple transfers across different treatment settings, such as from the hospital to rehabilitation centers, home, and sometimes back to the hospital. These transitions represent critical phases in their recovery process, yet they also pose a significant risk for medication errors and adverse events [11]. According to the National Hip Fracture Database, there has been a consistent decline in 30‐day mortality rates over a 10‐year period, dropping from 10.9% in 2007 to 6.7% in 2016 [12]. Further reductions in mortality could be achieved through the optimal implementation of recovery care strategies.

The functional outcomes following hip fracture surgery are influenced by a variety of factors [13]. Patients are at risk of experiencing diminished physical function [14, 15], an increased likelihood of falls and subsequent fractures [16], and a greater dependency on caregiving support [17]. Studies indicate that within 12 months of a hip fracture, approximately 50% of patients lose the ability to walk independently or perform daily activities without assistance. Additionally, around one‐third of older adults either become fully dependent on others or move to nursing homes within the first year following the fracture [18, 19].

Numerous organizations in high‐income countries have established clinical guidelines for hip fracture management [20, 21], highlighting the critical role of postoperative care in enhancing patient outcomes. Evidence indicates that interventions such as physiotherapy, occupational therapy, and effective pain management during the postoperative recovery phase significantly improve functional recovery. Additionally, these guidelines recommend standardized protocols to prevent complications, including pressure ulcers, deep vein thrombosis, and pulmonary embolism [22, 23]. Recent interventions have increasingly emphasized health education, health promotion, and the implementation of telehealth services [24].

Caregivers play a crucial role in the recovery of patients with hip fractures. However, research indicates that data on the characteristics of postdischarge care, especially during the transition to home, remain insufficient [25, 26]. The relatively short duration of hospital stays (13.5 ± 10.4 days) [15] often leaves patients more dependent on others for activities of daily living [25]. In most cases, the primary informal caregiver is a family member [27], who provides a cost‐effective alternative to formal postdischarge care services [25]. The term “family caregiver” is used to describe an individual who possesses a strong emotional connection to the patient, is a member of the patient′s family, and is able to provide both emotional support and caregiving throughout the course of the family member′s illness [28]. Understanding postoperative care practices is one of the most critical needs for caregivers of hip fracture patients. Key aspects such as the timing of suture removal, the level and type of physical activity permitted, guidelines for bathing, resuming sexual activity, and driving are particularly important in ensuring a safe and effective recovery [29]. Many of these care practices significantly influence patient rehabilitation following hip surgery [30].

### 1.1. Background

Within the KAP (knowledge, attitude, practice) model, it establishes knowledge as the basis for behavioral change, with attitudes serving as the key facilitators [31]. One of the applied fields of this model has been fracture care, where it has been used to assess and develop caregivers′ competencies [32]. Previous studies indicate that implementing interventions based on the KAP model can significantly improve fracture care outcomes. For instance, findings suggest that enhancing caregivers′ knowledge of first aid for fractures leads to improved immediate care practices and a reduction in complication rates [33]. Furthermore, within the realm of fracture management, this model has proven particularly effective in improving wound care practices and enhancing adherence to rehabilitation protocols [34]. Additionally, the improvement of performance among family caregivers necessitates the cultivation of a positive attitude and the expansion of their knowledge [34]. The evidence suggests a positive relationship between family caregivers′ KAP scores and care outcomes. This is demonstrated by the association of higher KAP scores with increased adherence to therapeutic guidelines and decreased levels of caregiver stress [31, 35].

The KAP theoretical framework serves as the foundation of this study. This model conceptualizes knowledge as the essential basis for informed decision‐making, attitude as the behavioral motivator, and practice as the tangible manifestation of these two components in the form of concrete actions. This framework guided both the design of the research questionnaire and the interpretation of variable relationships during the analysis phase.

According to this model, the behavior change process occurs in three sequential stages: knowledge acquisition, attitude formation, and finally, the emergence of new behaviors [36]. However, it is important to note that knowledge acquisition alone does not automatically lead to behavior change; rather, knowledge must first bring about a shift in perceptions and attitudes, which subsequently influences the individual′s behavior [37].

While the burden and challenges faced by these caregivers are well‐documented [25, 26], there is a notable scarcity of systematic evidence characterizing their specific levels of KAP in our local context. Without a baseline assessment of caregiver KAP, the development of targeted and effective educational interventions remains arbitrary. The initial and necessary step in designing any evidence‐based postoperative support program is, therefore, to first objectively evaluate the existing KAP status among this crucial group. Consequently, this study is aimed at investigating the knowledge, attitudes, and practices of family caregivers regarding home‐based care for patients with hip fractures (surgery for femoral neck or intertrochanteric fractures) in Kashan city in 2024.

## 2. Methods

The current study is a descriptive‐analytical research aimed at examining the KAP of family caregivers of patients with hip fractures in Kashan city during the year 2024.

### 2.1. Sampling and Sample Size

This study employed a convenience sampling method. The research population consisted of family caregivers of patients who had undergone surgery for a hip fracture at Shahid Beheshti Hospital in Kashan, Iran, during the data collection period (from May to August 2024). The inclusion criteria for the family caregivers were as follows: being the primary family caregiver responsible for the patient′s home care after discharge; having no physical or cognitive disabilities that would prevent participation in a telephone interview; expressing willingness to participate and providing verbal consent; and having sufficient time and willingness to respond to the questionnaire items.

The sample size was determined using a standard formula, with reference to a similar study [38]. Based on the parameters *d* = 0.5, Z1 − *α*/2 = 1.96, and *S* ^2^ = 14.65, the initial sample size was calculated to be 141 caregivers. To enhance the study′s statistical power and account for potential sample attrition, the final sample size was increased to 215. The formula used for calculating the sample size is provided below.

N=Z2∗S212−∝/ d2

The sampling process was conducted as follows: among the six hospitals in Kashan, three were identified as performing hip fracture surgeries. From these three, Beheshti Hospital in Kashan was chosen as the research site due to its higher number of patient referrals. Sampling of family caregivers of patients was conducted with the assistance of a hospital nurse. The study′s objectives and rationale were first explained to the nurse in detail. Following this, one of the researchers trained the nurse on how to ask questions and accurately record responses. A researcher‐designed questionnaire, developed to evaluate the KAP of family caregivers regarding postoperative hip surgery care, was then administered to the nurse. Using the ICD‐10 codes for hip fracture (S72.0, S72.1, S72.2, S72.3, S72.4, and S72.5), the nurse identified patients who had experienced a hip fracture within the past 2 years through the Sinai National Trauma Registry System. Subsequently, a list of these patients was extracted from the system.

Structured telephone interviews were employed for data collection. The interview was conducted after the caregiver had gained some experience in providing postoperative care at home, ensuring their responses were based on actual practice rather than hypothetical knowledge. Utilizing telephone interviews for data collection offers several significant advantages. This approach enables the efficient allocation of both financial and human resources. It also helps mitigate certain limitations inherent to face‐to‐face interview methods. Furthermore, the telephone interview format can foster a positive and productive rapport between the research team and participants. Ultimately, these factors collectively contribute to enhancing the overall quality and integrity of the data gathered [39].

A trained hospital nurse, who had received detailed instruction from the research team on the questionnaire and interview techniques, contacted caregivers. Upon contact, the nurse asked to speak with the primary family caregiver. The caregiver′s status was verified by asking two screening questions (a) their role in the patient′s direct daily care activities (e.g., moving, eating, or medication) and (b) their role as the patient′s primary care coordinator. Only individuals confirming this role proceeded to the next step. After obtaining verbal consent, the nurse administered the questionnaire by reading each question aloud to the participants and recording their responses directly onto the data collection sheet. This method was chosen to ensure consistency, facilitate participation from caregivers who might not be able to attend in‐person sessions, and increase the accessibility of the study for the target population. After completing the questionnaire, if the caregivers had any medical‐related questions, the nurse addressed them. The sampling and data collection process was conducted over a period of 4 months (from May to August 2024). This timeframe was necessary to systematically identify eligible patients from the registry, contact their caregivers, schedule the interviews, and complete the target of 215 questionnaires. The data collection tool used in this study was a researcher‐designed questionnaire consisting of four parts.

### 2.2. Part 1

#### 2.2.1. Personal and Contextual Information

The first section of the questionnaire comprised 11 demographic questions designed to assess the personal and contextual characteristics of family caregivers. These questions included: age, gender, caregiver‐patient familial relationship, education level, occupation, income, type of residence, marital status, caregiver nationality, history of training in patient care, and experience in caring for surgical patients.

### 2.3. Part 2

#### 2.3.1. Knowledge Construct

The second section consisted of 29 questions. This section assessed the caregivers′ understanding of factual information and essential medical guidelines related to postoperative hip care. The domains covered included: wound care—signs of infection, proper hygiene, and dressing management. Sample Knowledge questions include: “Using anti‐embolism stockings (compression stockings) is appropriate for a patient who has had surgery” and “ If a patient′s fever is over 38°, they should be given acetaminophen.”

Responses were recorded on a three‐point scale (true, false, do not know). A “true” response was assigned a score of 2, a “do not know” response was assigned a score of 1, and a “false” response was assigned a score of 0. The total possible score for this section ranged from 0 to 58, with 58 being the highest and 0 the lowest.

### 2.4. Part 3

#### 2.4.1. Attitude Construct

The third section comprised 20 questions and evaluated the caregivers feelings, perceptions, and beliefs toward the caregiving role. Sample attitude questions include: “In your opinion, should the patient be administered anticoagulant medications, such as Lovenox (enoxaparin) injections or aspirin?” and “In your opinion, should the patient′s active be significantly limited for the first two weeks postoperation?”

Responses were collected using a five‐point Likert scale ranging from *strongly disagree* (1) to *strongly agree* (5). The total score for the section ranged from 20 to 100, where 100 represented the highest possible score and 20 the lowest.

### 2.5. Part 4

#### 2.5.1. Practice Construct

This section measured the self‐reported application of caregiving activities. The domains covered included: application of care techniques, preventative measures, and adherence to guidelines. Sample practice questions include: “Whenever you bathed your patient, did you cover the dressing with a bag to keep it from getting wet?” and “Did you use an ice pack to prevent or reduce swelling in your patient′s foot?”

The fourth section included 26 questions designed on a four‐point Likert scale, ranging from “not at all” to “always”. Responses were scored as follows: “always” was assigned four points, whereas “not at all” was assigned one point. The total possible score for this section ranged from 26 to 104, with 104 representing the highest and 26 the lowest possible score.

### 2.6. Questionnaire Validity and Reliability

To ensure the quality of the research tool, the validity and reliability of the questionnaire were assessed through the following steps:

#### 2.6.1. Qualitative Face Validity

After the initial design of the questionnaire, 10 family caregivers were asked to review it and identify any items that were unclear or difficult to understand. Based on their feedback, certain medical terms that were challenging for the caregivers were simplified with the assistance of an orthopedic specialist.

#### 2.6.2. Qualitative Content Validity

The questionnaire was sent to 10 experts in relevant fields, including orthopedic specialists, nurses, and professionals in health education and health promotion. These experts evaluated the questionnaire in terms of wording difficulty, clarity, understandability, and the accuracy of the questions.

To assess quantitative content validity, two methods were employed: The content validity ratio (CVR) and the content validity index (CVI). The questionnaire was distributed to 10 specialists in fields such as medicine, nursing, orthopedics, epidemiology, and health education. The results were as follows: The CVR for the knowledge construct was 0.82, for attitude 0.79, and for the practice construct 0.80. The CVI for the knowledge construct was 0.78, for attitude 0.79, and for the practice construct 0.78. To determine the reliability of the instrument, the test‐retest method was used. The questionnaire was administered twice to 30 family caregivers with a 15‐day interval between administrations. The reliability coefficient obtained was 0.92, indicating excellent reliability of the research instrument.

### 2.7. Data Analysis

After coding, the questionnaire data were entered into SPSS Version 26 for analysis. The scoring metrics and total possible sums differed across the three constructs: knowledge (range: 0–58), attitude (range: 20–100), and practice (range: 26–104). To enable direct comparison and statistical analysis between these scales, all raw scores were converted into a standardized percentage of the maximum possible score (POMP) using the following formula:

POMP=Observed Score−Minimum Possible ScoreMaximum Possible Score−Minimum Possible Score×100.

This transformation resulted in comparable scores ranging from 0 to 100 for all three constructs, where higher values indicate more favorable outcomes. All subsequent analyses were performed using these normalized values. Descriptive statistics, including mean and standard deviation, were used to summarize the data. Inferential statistical methods, such as independent *t*‐tests, ANOVA, Pearson correlation, and linear regression, were applied to examine relationships and differences. A *p* value of less than 0.05 was considered statistically significant.

## 3. Results

According to the study findings, 69.8% of the caregivers were female, whereas 30.2% were male. Among the caregivers, 23% were spouses of the patients, 33.5% possessed a diploma, and 82.8% reported having an average income. Additionally, 41.9% of caregivers were aged between 42 and 51 years, 72.6% had prior experience in caring for the patient, and 86% of the 215 caregivers held Iranian citizenship (Table 1).

**Table 1:** Demographic variables information (*N* = 215).

**Variables**	**Categories**	**Frequency (** **n** **)**	**Percent (%)**
Caregiver’s age	20–30	26	12.1
	31–41	80	37.2
	42–51	90	41.9
	52–61	19	8.8

Caregivers gender	Female	150	69.8
	Male	65	30.2

Family relationship between caregiver and patient	Wife	50	23.3
	Mother	46	21.4
	Father	17	7.9
	Sister	14	6.5
	Brother	10	4.7
	Daughter	47	21.9
	Son	31	14.4

Caregiver education	Illiterate	9	4.2
	Elementary	35	16.3
	High School	52	24.2
	Diploma	72	33.5
	Bachelor	47	21.9

Caregiver job	Housewife	136	63.3
	Freelance	38	17.7
	Employee	31	14.4
	Unemployed	10	4.7

Caregiver′s income	Low	48	22.3
	Average	167	77.7
	High	0	0

Caregiver residence type	Landlord	147	68.4
	Tenant	68	31.6

Marital status of caregiver	Single	9	4.2
	Married	199	92.6
	Widow/Divorced	7	3.3

Caregiver nationality	Iranian	185	86.0
	Non‐Iranian	30	14.0

Educational background in caring for a patient with a hip fracture	Yes	160	74.4
	NO	55	25.6

History of care for a patient with a hip fracture	Yes	156	72.6
	NO	59	27.4

Table 2 displays the mean and standard deviation for the constructs of knowledge, attitude, and practice. The mean and standard deviation for attitude (84.82 ± 5.22) are higher than those for practice (78.65 ± 5.14) and knowledge (74.68 ± 6.29). The results of the Pearson correlation coefficient test indicate that the strongest correlation exists between practice and knowledge, which is both significant and direct (*R* = 0.422, *p* < 0.001) (Table 3).

**Table 2:** The mean scores of variables.

**Variables**	**M** **e** **a** **n** ± **S** **D**
Knowledge	74.68 ± 6.29
Attitude	84.82 ± 5.22
Practice	78.65 ± 5.14

**Table 3:** The correlation coefficient between the knowledge, attitude, and practice of family caregivers.

**Variables**	**Knowledge**	**Attitude**	**Performance**
Knowledge	1	—	—
Attitude	*R* = 0.311*p* < 0.001	1	—
Practice	*R* = 0.422*p* = 0.001	*R* = 0.148*p* = 0.030	1

The results of this study caregiver education were significantly associated with knowledge (*p* = 0.046) and attitude (*p* = 0.002), whereas caregiver occupation was significantly associated with both knowledge (*p* = 0.039) and attitude (*p* = 0.004). Caregiver income was significantly associated with knowledge (*p* = 0.002) and attitude (*p* = 0.004). Caregiver marital status showed a significant association only with attitude (*p* = 0.030). Additionally, caregiver nationality was significantly associated with knowledge (*p* < 0.001) and practice (*p* = 0.008). Furthermore, educational background in caring for a patient with a hip fracture was significantly associated with knowledge (*p* < 0.001) and attitude (*p* = 0.030), and patient care history was significantly associated with knowledge (*p* < 0.001) and attitude (*p* = 0.025) (Table 4).

**Table 4:** The relationship between demographic variables and knowledge, attitude, and practice in family caregivers.

**Variables**	**Categories**	**Knowledge** **m** **e** **a** **n** ± **S** **D**	**p** **value**∗	**Attitude** **m** **e** **a** **n** ± **S** **D**	**p** **value**∗	**Practice** **m** **e** **a** **n** ± **S** **D**	**p** **value**∗
Caregiver′s age	20–30	71.80 ± 6.78	0.078	85.19 ± 4.62	0.972	78.53 ± 4.77	0.653
	31–41	75.23 ± 6.69		84.82 ± 5.66		78.17 ± 5.61	
	42–51	74.77 ± 5.91		84.67 ± 4.66		79.16 ± 4.99	
	52–61	75.89 ± 4.67		85.05 ± 6.81		78.42 ± 4.31	

Caregivers gender	Female	74.36 ± 6.85	0.246	84.51 ± 5.27	0.181	78.84 ± 5.41	0.410
	Male	75.44 ± 4.69		85.55 ± 5.08		78.21 ± 4.46	

Family relationship between caregiver and patient	Wife	75.50 ± 8.10	0.147	85.78 ± 3.85	0.193	80.06 ± 5.01	0.060
	Mother	72.43 ± 6.22		83.54 ± 6.06		77.58 ± 6.36	
	Father	74.82 ± 4.68		86.88 ± 4.84		78.52 ± 5.54	
	Sister	73.50 ± 4.73		85.42 ± 4.70		76.42 ± 2.65	
	Brother	74.60 ± 5.60		85.70 ± 3.65		76.00 ± 5.12	
	Daughter	75.40 ± 5.91		84.02 ± 5.59		79.38 ± 4.88	
	Son	76.12 ± 4.46		84.74 ± 5.79		78.80 ± 3.49	

Caregiver education	Illiterate	71.00 ± 6.44	0.046	83.77 ± 4.84	0.002	78.22 ± 4.29	0.091
	Elementary	73.05 ± 6.23		83.68 ± 4.81		76.48 ± 4.83	
	High School	74.28 ± 7.06		83.48 ± 6.12		79.42 ± 5.72	
	Diploma	74.83 ± 5.95		84.86 ± 5.08		79.05 ± 5.15	
	Bachelor	76.63 ± 5.53		87.31 ± 3.83		78.89 ± 4.55	

Caregiver job	Housewife	74.02 ± 6.58	0.039	84.07 ± 5.18	0.004	78.77 ± 5.48	0.736
	Freelance	76.44 ± 5.13		85.18 ± 5.88		78.78 ± 4.82	
	Employee	76.29 ± 5.89		87.77 ± 3.53		77.74 ± 4.42	
	Unemployed	72.00 ± 5.39		84.60 ± 4.85		79.40 ± 3.59	

Caregiver′s Income	Low	72.25 ± 6.46	0.002	82.93 ± 6.62	0.004	75.91 ± 5.89	0.057
	Average	75.38 ± 6.07		85.37 ± 4.63		76.44 ± 5.89	
	High	0.00		0.00		0.00	

Caregiver residence Type	Landlord	74.82 ± 5.77	0.314	84.73 ± 5.55	0.702	78.41 ± 4.65	0.314
	Tenant	74.38 ± 7.31		85.02 ± 4.47		79.17 ± 6.06	

Marital status of caregiver	Single	74.88 ± 5.34	0.213	88.66 ± 2.54	0.030	78.88 ± 4.28	0.422
	Married	74.82 ± 6.33		84.75 ± 5.13		78.73 ± 5.18	
	Widow/divorced	70.57 ± 5.41		82.00 ± 8.04		76.14 ± 4.70	

Caregiver nationality	Iranian	75.42 ± 6.00	0.000	85.10 ± 5.36	0.051	79.02 ± 5.07	0.008
	Non‐Iranian	70.13 ± 6.21		83.10 ± 3.94		76.36 ± 5.03	

Educational background in caring for a patient with a hip fracture	Yes	75.54 ± 6.05	0.001	85.28 ± 4.91	0.030	78.67 ± 5.07	0.926
	NO	72.20 ± 6.34		83.50 ± 5.89		78.60 ± 5.38	

History of care for a patient with a hip fracture	Yes	71.06 ± 6.53	0.000	85.31 ± 5.36	0.026	78.41 ± 5.07	0.256
	NO	76.05 ± 5.63		83.54 ± 4.63		79.30 ± 5.29	

∗Independent *t*‐test, ANOVA.

A linear regression model was used to examine the influence of knowledge, attitude, and significant demographic variables (identified in univariate analyses) on the practice variable. As shown in Table 5, the final model identified knowledge (*β* = 0.289, *p* < 0.001), Iranian nationality (*β* = −0.223, *p* = 0.001), and a history of caregiving (*β* = 0.191, *p* = 0.004) as significant predictors of practice. Collectively, these three variables explained approximately 31% of the variance in the practice variable (*R* ^2^ = 0.312).

**Table 5:** Factors predicting practice based on knowledge, attitude, and demographic factors.

**Variables**	**Unstandardized coefficients**		**Standardized coefficients**	**t**	**p** **value**
	**B**	**Std. error**	**Beta**		
(Constant)	60.975	4.201	—	14.513	0.000
Knowledge	0.236	0.052	0.289	4.552	0.000
Caregiver nationality	−3.306	0.953	−0.223	−3.470	0.001
History of caregiving	2.195	0.757	0.191	2.901	0.004
Adjusted *R*square = 0.312					

## 4. Discussion

The present study is aimed at exploring the knowledge, attitude, and practice of family caregivers regarding caregiving behaviors following hip surgery at Shahid Beheshti Hospital in Kashan. The mean scores revealed that caregivers held positive attitudes, followed by practice and knowledge. This aligns with studies by Chen et al. and Elnaghy et al. in the context of home‐based cancer care and cerebral infarction, which suggest that a willingness to care often precedes deep knowledge or optimal practice [40, 41].

The strongest correlation was observed between knowledge and practice (*R* = 0.422, *p* < 0.001), underscoring that increased understanding of postoperative care directly translates into better caregiving behaviors. This finding is consistent with fundamental educational principles and prior research [41, 42], which demonstrate that enhancing knowledge through structured training is a critical step toward improving practical skills and behavioral outcomes in caregiving.

This finding aligns with the study by Gray et al., which demonstrated that positive caregiver attitudes contribute to enhanced care quality and reduced stress levels [43]. Additionally, caregivers′ attitudes were found to be influenced by factors such as gender, marital status, and income, a pattern consistent with findings from other studies [44–46].

However, the novel contribution of our study lies in the regression analysis, which moves beyond established correlations to identify key predictors. We found that knowledge, Iranian nationality, and a history of caregiving collectively explained 31% of the variance in caregiving practice. The positive influence of knowledge reinforces the need for structured education. More importantly, the significant predictive value of Iranian nationality points to the profound role of cultural context. This may be attributed to culturally ingrained filial piety, familiarity with the Iranian healthcare system, and a shared language facilitating clearer communication with healthcare providers, all of which enhance caregiving efficacy. These findings align with previous studies, which indicate that caregivers′ knowledge is often influenced by factors such as education level, access to educational resources, and prior caregiving experience [42, 47]. Adequate knowledge and experience can enhance the self‐efficacy of the patient′s family, enabling them to provide more effective care after returning home [48]. Liu et al. reported an increase in health‐related knowledge at the time of patient discharge, attributing this improvement to the effective delivery of health information through educational care programs. However, they emphasized that these programs alone are insufficient to foster sustainable changes in health‐promoting behaviors [49].

Furthermore, the positive impact of a history of caregiving highlights the importance of experiential learning. Caregivers with prior experience likely possess improved problem‐solving skills, lower anxiety, and greater confidence in managing patient needs, which cannot be fully replaced by theoretical training alone [48]. This finding is particularly novel as it quantifies the value of lived experience in a caregiving model.

Interestingly, while attitude scores were high, they were not a significant predictor of practice in the multivariate model. This suggests that in this population, practical capability (knowledge and experience) and cultural context may be more powerful drivers of behavior than positive intentions alone. This nuance adds a critical layer to the interpretation of KAP studies, indicating that high attitude scores should not be misinterpreted as a guarantee of high‐quality care without the necessary knowledge and enabling environment.

Also, caregiver practice, with a mean score of 78.65 (±5.14), indicated a relatively favorable level of caring behaviors. This finding aligns with the studies by Chen et al. and Elnaghy et al., which demonstrated that caregiver practice is directly influenced by their knowledge and attitudes [40, 41].

Despite its valuable findings, this study has several limitations that should be considered when interpreting the results. Firstly, the use of convenience sampling from a single hospital may introduce selection bias and limit the generalizability of the findings to the broader population of family caregivers in other regions or healthcare settings. Secondly, the reliance on a self‐reported questionnaire for data collection may be subject to recall bias and social desirability bias, particularly concerning caregivers′ reported practices. Thirdly, the cross‐sectional design of the study prevents the establishment of causal relationships between the variables examined. Fourthly, construct validity assessment for the questionnaire was not conducted. Therefore, future researchers intending to use this questionnaire in different research contexts or with different populations should perform a construct validity assessment to ensure the instrument′s validity and generalizability in the new setting. Finally, the strong predictive role of Iranian nationality, while insightful, may reflect context‐specific cultural factors that limit the extrapolation of this finding to other cultural or national settings. Future studies should consider employing longitudinal designs, multicenter randomized sampling, and mixed‐methods approaches to overcome these limitations and provide a more comprehensive understanding of the factors influencing caregiver practices.

### 4.1. Clinical Implications and Recommendations

The findings of this study have several direct implications for clinical practice and the development of support systems for family caregivers: implement structured, hands‐on pre‐discharge training programs—Since knowledge was the strongest modifiable predictor of practice, hospitals must move beyond ad hoc advice to standardized education sessions. We recommend developing a compact, multidisciplinary training module for caregivers before patient discharge. This module should be available in both Farsi and other prevalent languages. It must cover practical skills: wound care, safe patient mobilization and transfer techniques, medication management, recognition of red‐flag complications (e.g., signs of infection or deep vein thrombosis), and nutritional guidance. Utilizing teach‐back methods and simple visual aids can ensure comprehension and retention.

Develop targeted support for inexperienced and non‐Iranian caregivers: Our evidence shows that caregivers without prior experience or of non‐Iranian nationality are at a significant disadvantage. Clinics and hospitals should identify these vulnerable caregivers during admission. We recommend assigning a nurse case manager (an experienced caregiver) to provide them with intensified, repeated training and a direct phone line for postdischarge consultations. This tailored support can help bridge the gap caused by a lack of experience or cultural/linguistic barriers.

Follow‐up Mechanism: The care relationship should not end at discharge. We recommend implementing a structured telephone follow‐up system at 24–48 h, 1 week, and 2 weeks postdischarge. This proactive contact allows healthcare professionals to assess the patient′s status, answer caregiver questions, reinforce training, and provide psychological support, ultimately improving care quality and reducing caregiver burden.

## 5. Conclusion

In conclusion, this study demonstrates that effective home care after hip surgery is significantly influenced by the caregiver′s knowledge, cultural context, and prior experience. Merely having a positive attitude is insufficient without the requisite skills and support. The novel contribution of this work is the quantification of these specific demographic and experiential factors within an Iranian context. To translate these findings into action, we strongly recommend the development of standardized, hands‐on training programs that are particularly targeted toward inexperienced and non‐Iranian caregivers. Furthermore, establishing structured postdischarge follow‐up protocols is essential to sustain care quality. Investing in caregiver education and support is not an ancillary activity but a fundamental component of successful orthopedic patient recovery, ultimately leading to reduced complications, improved functional outcomes, and lower healthcare costs.

**Nomenclature**
CVR: content validity ratio
CVI: content validity index
KAP: knowledge, attitude, and practice

## Ethics Statement

This research adhered to ethical considerations and principles, with the required approval granted by the Research Vice‐Chancellor of Kashan University of Medical Sciences (Ethical Code: IR.KAUMS.MEDNT.REC.1403.064).

## Consent

Informed consent was obtained from all the participants. There was an emphasis on maintaining privacy in keeping and delivering the information accurately without mentioning the names of the participants.

## Conflicts of Interest

The authors declare no conflicts of interest.

## Author Contributions

S.Y.: conceptualization. M.V. and F.S.: data curation. A.J.: formal analysis. E.F., M.S. and S.Y.: investigation. M.M. and K.K.: methodology. S.Y.: project administration. M.R.F. and M.A‐K.: supervision. A.A.: visualization. S.Y.: writing—original draft. E.F., M.S., A.J., M.R.F., M.M., K.K., A.A., M.A‐K. and S.Y.: writing—review and editing.

## Funding

No funding was received for this manuscript.

## Data Availability

The data that support the findings of this study are available on request from the corresponding author. The data are not publicly available due to privacy or ethical restrictions.
